# DVH- and NTCP-based dosimetric comparison of different longitudinal margins for VMAT-IMRT of esophageal cancer

**DOI:** 10.1186/s13014-017-0871-3

**Published:** 2017-08-15

**Authors:** S Münch, M Oechsner, SE Combs, D Habermehl

**Affiliations:** 1 Department of Radiation Oncology, Klinikum rechts der Isar, TU München, Ismaninger Str. 22, 81675 Munich, Germany; 2 German Cancer Consortium (DKTK), Partner Site Munich, Munich, Germany; 3 Institute of Innovative Radiotherapy (iRT), Helmholtz Zentrum München, Ingolstädter Landstraße 1, 85764 Neuherberg, Oberschleißheim Germany

**Keywords:** Esophageal cancer, Modern radiation techniques, Longitudinal margins, Dosimetric effects

## Abstract

**Purpose:**

To cover the microscopic tumor spread in squamous cell carcinoma of the esophagus (SCC), longitudinal margins of 3–4 cm are used for radiotherapy (RT) protocols. However, smaller margins of 2–3 cm might be reasonable when advanced diagnostic imaging is integrated into target volume delineation. Purpose of this study was to compare the dose distribution and deposition to the organs at risk (OAR) for different longitudinal margins using a DVH- and NTCP-based approach.

**Methods:**

Ten patients with SCC of the middle or lower third were retrospectively selected. Three planning target volumes (PTV) with longitudinal margins of 4 cm, 3 cm and 2 cm and an axial margin of 1.5 cm to the gross target volume (GTV) were defined for each patient. For each PTV two treatment plans with total doses of 41.4 Gy (neoadjuvant treatment) and 50.4 Gy (definite treatment) were calculated. Dose to the lungs, heart, myelon and liver were then evaluated and compared between different PTVs.

**Results:**

When using a longitudinal margin of 3 cm instead of 4 cm, all dose parameters (Dmin, Dmean, Dmedian and V5-V35), except Dmax could be significantly reduced for the lungs. Regarding the heart, a significant reduction was seen for Dmean and V5, but not for Dmin, Dmax, Dmedian and V10-V35. When comparing a longitudinal margin of 4 cm to a longitudinal margin of 2 cm, a significant difference was calculated for Dmin, Dmean, Dmedian and V5-V35 of the lungs and for Dmax, Dmean and V5-V35 of the heart. Nevertheless, no difference was seen for median heart dose. An additional dose reduction for V10 of the heart was achieved for definite treatment plans when using a longitudinal margin of 3 cm. The NTCP-based risk of pneumonitis was significantly reduced by a margin reduction to 2 cm for neoadjuvant and definite treatment plans.

**Conclusion:**

Reduction of longitudinal margins from 4 cm to 3 cm can significantly reduce the dose to lungs and Dmean of the heart. Despite clinical benefit and oncologic outcome remain unclear, reduction of the longitudinal margins might provide the opportunity to reduce side effects of chemoradiation (CRT) for SCC in upcoming studies.

## Introduction

Esophageal cancer (EC) is the eighth most common cancer in the world and more than 450.000 new cases are diagnosed each year [1]. Since several studies demonstrated the advantage of neoadjuvant chemoradiation (nCRT) compared to surgery alone and therefore multimodal therapy became the treatment of choice for patients with locally advanced squamous cell carcinoma of the esophagus (SCC) [2–4]. In addition, definite chemoradiation (dCRT) is recommend for patients unsuitable for surgery or if surgery is refused by the patient [5, 6]

One of the most important challenges when planning radiotherapy for EC are microscopic tumor spreads that are not visible by computer tomography and therefore complicate the definition of the clinical target volume (CTV). An analysis of data from surgical resections identified microscopic intraepithelial and/ or subepithelial tumor extensions in 46% and 55% of patients, respectively, with less than 5% risk of positive resection margins at 3 cm [7].

To cover these areas that are at higher risk for microscopic tumor spread, radiotherapy (RT) for EC is typically done with large longitudinal safety margins, which lead to a significant dose exposure to the organs at risk (OAR).

While Herskovic et al. [6] even irradiated the whole esophagus in an early trial, two later studies used cranio-caudal margins of 5 cm [8, 9]. In the most recent study by van Hagen et al. [10], which defined a new standard for nCRT in EC patients, the Planning Target Volume (PTV) was defined by adding longitudinal margins of 4 cm to the GTV. Today, when planning a neoadjuvant or dCRT for SCC the German S3-guideline [11] as well as the North American guidelines [5] recommend to define the clinical target volume (CTV) by adding a longitudinal safety margin of 3 cm to 4 cm and an axial margin of 1 cm to 1.5 cm to the GTV. To consider inter-fractional movement and anatomic changes due to breathing, another margin of 0.5–1.5 cm should then be added to the CTV.

Aim of this study is to evaluate the dosimetric effects of reduced longitudinal margins for nCRT and dCRT of SCC.

## Methods

### Patient Characteristics

For this study, we evaluated treatment plans with different longitudinal margins for 10 patients with thoracic SCC. All patients had T2–3 N0–1 carcinoma. Patients´ characteristics are shown in Table 1.

**Table 1:** Patients’ characteristics

Patients’ characteristics	Number of cases (%)	Median (IQR)
Tumor extension in cm		6.0 (5.0–6.5)
PET-positive lymph nodes	*n* = 8 (80%)	
Volume of GTV in cm^3^		20.3 (13.0–28.0)
Volume of PTV4 in cm^3^		387.2 (323.9–523.9)
Volume of PTV3 in cm^3^		349.3 (291.3–472.5)
Volume of PTV2 in cm^3^		310.5 (252.7–419.3)

*GTV* (Gross tumor volume), *PTV* (Planning target volume), *IQR* (interquartile range)

The primary tumor was delineated using all available diagnostic information (esophago-gastro-duodenoscopy, endoscopic ultrasound (EUS) and positron emission tomography (PET)). Lymph nodes were considered as metastatic if PET showed an increased glucose uptake. Primary tumor and Lymph-node metastases were defined as the GTV. If the planning CT was performed as a 4-dimensional CT, we also delineated an internal target volume (ITV). Analogous to the CROSS trial a CTV was not routinely in this study. Instead, the GTV/ ITV was extended by an axial margin of 1.5 cm and longitudinal margins of 2 cm, 3 cm and 4 cm. These expanded GTVs/ ITVs were than adapted according to the individual expertise of the treating radiation oncologist to generate the final PTVs with longitudinal margins of 2 cm (PTV 2), 3 cm (PTV 3) and 4 cm (PTV 4). Thereby, the individual axial safety margin was the same for each longitudinal margin (PTV2–4).

The lungs, the heart, the liver and the myelon were delineated manually.

### Treatment Planning

For all ten patients a neoadjuvant and a definite treatment plan was calculated for each PTV with the Eclipse 13.0 planning system (Varian Medical Systems, Palo Alto, CA, USA). Volumetric-modulated arc therapy (VMAT) plans with two full arcs (358° rotation) were optimized. Dose calculation was performed using the Anisotropic Analytical Algorithm (AAA) and heterogeneity correction. The prescribed dose for the neoadjuvant treatment plans was 41.4 Gy with single doses of 1.8 Gy per fraction and 50.4 Gy with single doses of 1.8 Gy per fraction for the definite treatment plans. All plans were normalized so that the median dose of the PTV corresponds to the prescription dose (41.4Gy or 50.4Gy). The planning goal was to achieve a homogeneous dose distribution within the PTV and to reduce the dose to OARs, in particular the lungs, heart, liver and myelon. To compare dose distribution to the OARs we analyzed absolute (mean dose (Dmean), median dose (Dmedian), minimum dose (Dmin) and maximum dose(Dmax)) and relative dose parameters (Volume receiving 5Gy, 10Gy, 15Gy, 20Gy, 25Gy, 30Gy and 35Gy (V5-V35)) for all treatment plans. The volumes receiving 40 Gy and 45 Gy (V40, V45) of the lungs and the heart were additionally evaluated for dCRT treatment plans.

### NTCP-Calculation

To estimate the clinical effects of the dose differences between the treatment plans the normal tissue complication probability (NTCP) was calculated for the whole lung and the heart. NTCP was calculated using the Niemierko model [12]. NTCP calculations were performed with Matlab software (the MathWorks inc, Natic, Maryland, USA) using a Matlab program provided by Gay HA et al. [13]. For that purpose the dose volume histogram for the heart and the whole lung was transferred from the TPS to Matlab in tabular form and NTCP values were calculated for all treatment plans for every patient.

### Statistics

Statistical calculations were performed using SPSS 18.0 software (SPSS Inc., Chicago, IL, USA). The distribution of quantitative data is described by median and interquartile range (IQR). Likewise, qualitative data is presented by absolute and relative frequencies. Statistical hypothesis testing was performed through t-test for paired samples. Statistical significance was considered at a *p*-value <0.05.

## Results

### Neoadjuvant treatment

Dose parameters of the OAR for neoadjuvant treatment and different longitudinal margins can be seen in Table 2. In order to evaluate the dosimetric impact of reduced longitudinal margins, a longitudinal safety margin of 4 cm was compared to longitudinal margins of 3 cm and 2 cm.

**Table 2:** Dose parameters for neoadjuvant treatment

Neoadjuvant treatment (41.4 Gy)					
	PTV2	PTV3	PTV4		
	Mean ± SD	Mean ± SD	Mean ± SD	*p*-Value^a^	*p*-Value^b^
Myelon					
Dmax (Gy)	31.65 ± 4.29	31.99 ± 4.02	32.68 ± 4.13	**0.036**	0.173
Liver					
Dmean (Gy)	0.50 ± 1.01	0.71 ± 1.33	0.95 ± 1.54	**0.020**	**0.033**
Dmedian (Gy)	0.18 ± 0.20	0.29 ± 0.39	0.49 ± 0.85	0.203	0.179
Heart					
Dmin (Gy)	0.91 ± 2.27	0.97 ± 2.20	1.03 ± 1.96	0.591	0.393
Dmax (Gy)	25.95 ± 19.04	35.54 ± 13.99	41.25 ± 5.98	0.118	**0.021**
Dmean (Gy)	6.48 ± 5.91	7.83 ± 6.27	8.83 ± 6.13	**0.036**	**0.009**
Dmedian (Gy)	4.95 ± 5.19	6.11 ± 5.85	6.47 ± 5.86	0.256	0.059
V5 (%)	35.81 ± 37.94	45.22 ± 38.64	53.14 ± 39.64	**0.002**	**0.001**
V10 (%)	26.15 ± 29.53	33.25 ± 31.89	37.34 ± 30.39	0.174	**0.016**
V15 (%)	13.94 ± 15.65	17.77 ± 17.00	19.59 ± 15.83	0.270	**0.023**
V20 (%)	7.97 ± 9.09	10.21 ± 9.85	11.20 ± 9.16	0.295	**0.027**
V25 (%)	5.05 ± 5.85	6.48 ± 6.30	7.18 ± 5.99	0.238	**0.026**
V30 (%)	3.47 ± 4.08	4.43 ± 4.37	5.03 ± 4.30	0.135	**0.021**
V35 (%)	2.50 ± 3.01	3.16 ± 3.23	3.69 ± 3.29	0.068	**0.018**
Lungs					
Dmin (Gy)	0.19 ± 0.12	0.24 ± 0.16	0.32 ± 0.22	**0.007**	**0.003**
Dmax (Gy)	42.45 ± 0.19	42.48 ± 0.28	42.62 ± 0.52	0.357	0.340
Dmean (Gy)	9.28 ± 1.85	10.33 ± 1.83	11.02 ± 1.86	**<0.001**	**<0.001**
Dmedian (Gy)	6.89 ± 3.92	8.51 ± 3.41	9.60 ± 2.46	**0.011**	**0.001**
V5 (%)	61.73 ± 17.57	69.24 ± 17.61	74.24 ± 18.25	**<0.001**	**<0.001**
V10 (%)	42.74 ± 9.29	48.43 ± 9.67	52.59 ± 10.47	**0.003**	**<0.001**
V15 (%)	20.84 ± 6.11	23.55 ± 6.37	25.42 ± 5.72	**0.004**	**<0.001**
V20 (%)	9.50 ± 3.51	10.70 ± 3.67	11.41 ± 3.45	**0.011**	**<0.001**
V25 (%)	4.68 ± 1.64	5.31 ± 1.70	5.64 ± 1.60	**0.017**	**<0.001**
V30 (%)	2.69 ± 0.75	3.05 ± 0.76	3.25 ± 0.72	**0.012**	**<0.001**
V35 (%)	1.74 ± 0.40	1.95 ± 0.39	2.08 ± 0.40	**0.017**	**<0.001**

^a^4 cm margin vs. 3 cm margin, ^b^ 4 cm margin vs. 2 cm margin, Gy Gray, cm centimeters

For the lungs, all dose parameters except Dmax were significantly reduced by shorter margins.

Regarding the heart, a significant dose reduction was seen for Dmean and V5 when comparing longitudinal margins of 4 cm to longitudinal margins of 3 cm. In contrast to that, no significant difference was seen for Dmin, Dmax, Dmedian and V10 - V35. When using longitudinal margins of 2 cm a significant dose reduction was observed for Dmean, Dmax and V5-V35, whereas there was no significant difference for Dmin and Dmedian. While Dmean of the liver was significantly reduced by longitudinal margins of 3 cm and 2 cm, there was no significant difference for Dmedian. In contrast to that, a significant reduction of the Dmax of the myelon was seen for longitudinal margins of 3 cm. However, this difference was not significant when comparing longitudinal margins of 4 cm to longitudinal margins of 2 cm.

### Definite treatment

Results for definite treatment plans and different longitudinal margins can be seen in Table 3.

**Table 3:** Dose parameters for definite treatment

Definite treatment (50.4 Gy)					
	PTV2	PTV3	PTV4		
	Mean ± SD	Mean ± SD	Mean ± SD	*p*-Value^*a*^	*p*-Value^b^
Myelon					
Dmax (Gy)	36.49 ± 3.09	37.21 ± 3.04	37.77 ± 3.05	0.146	0.130
Liver					
Dmean (Gy)	0.61 ± 1.24	0.85 ± 1.62	1.16 ± 1.88	**0.017**	**0.032**
Dmedian (Gy)	0.22 ± 0.25	0.34 ± 0.48	0.59 ± 1.04	0.196	0.179
Heart					
Dmin (Gy)	1.10 ± 2.76	1.18 ± 2.68	1.25 ± 2.39	0.557	0.384
Dmax (Gy)	31.48 ± 23.31	40.93 ± 17.28	50.23 ± 7.33	0.056	**0.021**
Dmean (Gy)	7.65 ± 7.41	9.26 ± 7.86	10.73 ± 7.48	**0.015**	**0.003**
Dmedian (Gy)	6.02 ± 6.33	7.36 ± 7.16	7.86 ± 7.14	0.212	0.059
V5 (%)	37.48 ± 38.56	45.89 ± 40.15	55.13 ± 39.55	**<0.001**	**0.001**
V10 (%)	31.27 ± 35.49	37.71 ± 37.10	45.18 ± 36.98	**0.008**	**0.007**
V15 (%)	19.52 ± 21.85	24.16 ± 24.36	27.50 ± 22.24	0.163	**0.020**
V20 (%)	11.76 ± 13.25	14.52 ± 14.79	16.46 ± 13.39	0.181	**0.024**
V25 (%)	7.55 ± 8.63	9.34 ± 9.62	10.60 ± 8.71	0.179	**0.027**
V30 (%)	5.20 ± 6.02	6.44 ± 6.68	7.37 ± 6.15	0.145	**0.026**
V35 (%)	3.78 ± 4.43	4.67 ± 4.89	5.45 ± 4.64	0.089	**0.022**
V40 (%)	2.87 ± 3.41	3.52 ± 3.75	4.20 ± 3.67	**0.046**	**0.019**
V45 (%)	2.19 ± 2.68	2.67 ± 2.93	3.23 ± 2.95	**0.030**	**0.018**
Lungs					
Dmin (Gy)	0.22 ± 0.15	0.29 ± 0.20	0.39 ± 0.27	**0.004**	**0.002**
Dmax (Gy)	50.79 ± 2.99	51.72 ± 0.40	51.80 ± 0.67	0.711	0.313
Dmean (Gy)	11.10 ± 2.48	12.39 ± 2.27	13.37 ± 2.27	**<0.001**	**<0.001**
Dmedian (Gy)	8.31 ± 4.86	9.96 ± 4.16	11.65 ± 3.00	**0.004**	**0.001**
V5 (%)	63.61 ± 18.19	70.52 ± 18.49	76.20 ± 18.29	**<0.001**	**<0.001**
V10 (%)	50.67 ± 12.94	57.46 ± 13.30	63.33 ± 14.45	**<0.001**	**<0.001**
V15 (%)	30.32 ± 8.43	35.01 ± 7.59	38.12 ± 7.26	**0.001**	**<0.001**
V20 (%)	15.96 ± 5.76	18.42 ± 5.35	20.04 ± 4.95	**0.001**	**<0.001**
V25 (%)	8.33 ± 3.38	9.62 ± 3.26	10.37 ± 3.11	**0.004**	**<0.001**
V30 (%)	4.65 ± 1.77	5.42 ± 1.75	5.83 ± 1.63	**0.008**	**0.001**
V35 (%)	2.87 ± 0.87	3.38 ± 0.90	3.63 ± 0.83	**0.009**	**0.001**
V40 (%)	1.90 ± 0.56	2.30 ± 0.50	2.47 ± 0.48	**0.011**	**0.011**
V45 (%)	1.23 ± 0.50	1.56 ± 0.30	1.68 ± 0.32	**0.027**	**0.046**

^a^4 cm margin vs. 3 cm margin, ^b^ 4 cm margin vs. 2 cm margin, Gy Gray, cm centimeters

Dose distribution to the OARs was higher for definite treatment plans than for neoadjuvant treatment plans. When comparing longitudinal margins of 4 cm to longitudinal margins of 3 cm and 2 cm, again a significant reduction of all dose parameters except the Dmax was seen for the lungs. In addition to Dmean and V5 a significant reduction was seen for V10, V40 and V45 of the heart when comparing a longitudinal margin of 4 cm to a longitudinal margin of 3 cm in definite treatment plans. When the longitudinal margin was reduced to 2 cm, there was a significant reduction of all dosimetric parameters except Dmin and Dmedian. Comparable to the neoadjuvant treatment plans a significant reduction was seen for Dmean but not for Dmedian of the liver. In addition, there was also no significant difference for the Dmax of the myelon.

### NTCP

The probability of normal tissue complications for the heart (pericarditis) and the lungs (pneumonitis) are demonstrated in Table 4.

**Table 4:** NTCP for neoadjuvant treatment (41.4Gy) and definite treatment (50.4 Gy)

	PTV2	PTV3	PTV4		
	Mean ± SD	Mean ± SD	Mean ± SD	*p*-Value^a^	*p*-Value^b^
Neoadjuvant treatment (41.4 Gy)					
Risk of pericarditis (%)	0.00 ± 0.00	0.01 ± 0.00	0.01 ± 0.00	0.183	0.169
Risk of pneumonitis (%)	0.02 ± 0.03	0.04 ± 0.06	0.06 ± 0.08	0.059	**0.031**
Definite treatment (50.4 Gy)					
Risk of pericarditis (%)	0.00 ± 0.01	0.01 ± 0.02	0.01 ± 0.02	0.111	0.172
Risk of pneumonitis (%)	0.10 ± 0.16	0.18 ± 0.26	0.29 ± 0.37	0.063	**0.032**

^a^Margin 4 cm vs. Margin 3 cm, ^b^ Margin 4 cm vs. Margin 2 cm

No significant differences were observed for the risk of pericarditis when comparing longitudinal margins of 4 cm to longitudinal margins of 3 cm or 2 cm. In contrast to that there was a significant difference for the risk of pneumonitis (0.06% vs. 0.02%; *p* = 0.031 and 0.29% vs 0.10%; *p* = 0.032) between longitudinal margins of 4 cm and longitudinal margins of 2 cm for neoadjuvant and definite treatment plans. In addition, there was also a strong trend for a decreased risk of developing any kind of pneumonitis when longitudinal margins of 4 cm were compared to longitudinal margins of 3 cm.

## Discussion

Chemoradiation plays an important role within multimodal treatment concepts for advanced SCC. While nCRT is considered the standard of care for patients with locally advanced SCC, dCRT is the only curative therapeutic option for patients unsuitable for surgery [5, 6, 10, 14].

When defining CTV and PTV a compromise has to be made between the risk of local treatment failure and the risk of treatment-related toxicities. Thereby the CTV has to include the primary tumor and lymph node metastases. In case of SCC, there are two uncertainties necessitate large cranio-caudal safety margins. The first one is, that identification of the macroscopic primary tumor with CT is often difficult and reveals a high inter-observer variability [15]. However, in recent years modern imaging techniques like PET/CT or magnetic resonance imaging have demonstrated their potential to improve tumor staging and target delineation for SCC [16–19] and therefore longitudinal safety margins have already been reduced in the past [6, 8, 10]. This is especially important because in contrast to other tumor entities no elective nodal irradiation (ENI) is performed for SCC [20, 21] and therefore, the extension of the primary tumor is directly associated with the extension of the CTV and the PTV.

The second uncertainty is caused by microscopic tumor spread. In esophageal cancer, microscopic, subepithelial tumor extension along the esophagus is commonly observed. However, a randomized phase-III trial by Gao et al. [22] demonstrated that the longitudinal microscopic tumor spread is smaller than 3 cm in more than 94% of patients. Furthermore, a study by Button et al. [23] who used EUS for the definition of the GTV, revealed that the rate of local failure after definite chemoradiation with longitudinal margins of 3 cm was similar to other studies, using larger longitudinal margins. In this study, only three patients (2%) had a tumor recurrence adjacent to the radiation field. Despite this limited results reduced longitudinal margins seem to be feasible in neoadjuvant or definite chemoradiation for SCC and In the future modern imaging-techniques might help to identify tumor extensions and offer the opportunity to reduce the longitudinal margins in the clinical routine.

While the combination of PET−/ and CT-imaging leads to a very high diagnostic sensitivity of 93% [24] and is able to detect unexpected distant metastases [25], PET has a low spatial resolution which impedes the detection of small, early stage carcinomas and limits the evaluation of the depth of invasion (T-stage) [26]. The low spatial resolution of PET also compromises the detection of peritumoural lymph node metastases and benign inflammation can lead to false-positive results in both the esophagus and the lymph nodes [25, 27]. In contrast to that, EUS is recommended to define T-stage. Comparative analyses demonstrated the superiority of EUS over PET/CT in terms of T-staging [28], while no significant difference was found between PET/CT and EUS regarding the accuracy for lymph node metastases [29]. However, both modalities are not appropriate to detect small or occult lymph node metastases [30, 31]. Distant lymph node metastases can be seen in up to 37% of patients which is especially important because ENI is not routinely done in SCC patients [32]. On the other hand, a large body of literature highlights the use of ENI which does not alter the oncological outcome in SCC patient undergoing radiotherapy [20, 33, 34].

In this planning-study, we analyzed the dosimetric impact of reduced longitudinal margins for patients treated with nCRT or dCRT. While a reduced longitudinal margin of 3 cm for nCRT leads to a significant reduction of almost all dose parameters of the lungs, a significant dose reduction was only determined for Dmean and V5 of the heart. It is most likely that this difference is based on the anatomical location. In contrast to the lungs, the heart is more exposed when the target volume reaches the lower part of the thorax and therefore dose distribution strongly depends on the localization and extension of the tumor. Therefore, relatively high inter-individual deviations and intra-individual differences are unlikely to show significant differences. This fact is also underlined by the high standard deviations, which are almost as high as the mean values for most dose parameters of the heart. Nevertheless, when comparing a halved longitudinal margin of 2 cm to a longitudinal margin of 4 cm the absolute intra-individual differences become higher and therefore a statistic significant reduction of most dose parameters is determined for the heart. The fact that a decrease of the median doses to the heart and the liver has not reached significance even when reducing the longitudinal margin to 2 cm can also be explained by this.

Our results determine that even a reduction of the longitudinal margin of just 1 cm can significantly reduce the dose to the lungs. However, absolute differences are small and clinical relevance should be discussed critically.

At first, we have to point out that clinical impacts were just analyzed theoretical by using the Niemierko NTCP-model [12]. Despite the problems with this approach, it was the only method to evaluate side effects for reduced margins, because at the moment all patients are treated analogue to the CROSS-protocol. However, with a focus to the lungs, several studies demonstrated that mean lung dose and different dose-volume histogram (DVH) parameters like V20 or V30 are associated with the risk of developing a radiation pneumonitis [35, 36]. In an analysis by Kumar et al. [35] mean V20 for the lungs was 24.9% for patients treated with intensity modulated radiotherapy and 45% of these patients had acute symptomatic pneumonitis. Despite V20 in these patients was higher than in our study, there is a remarkable difference between the incidence of pneumonitis in the analysis be Kumar et al. and the risk of pneumonitis as calculated by the NTCP model. It was shown before, that the inclusion of non-dosimetric risk factors can modify the results calculated by NTCP models [37]. Therefore, another explanation might be that our results underestimated the actual risk of radiation pneumonitis depending on further non-dosimetric risk factors. For example, one of these additional risk factors is the simultaneous chemotherapy. In a study by Shi et al. [38] that analyzed 94 patients with non-small cell lung cancer treated with simultaneous chemoradiation, V10 of the lungs was independently associated with the risk of radiation pneumonitis. Thereby, the risk of severe radiation pneumonitis was 5.7% in patients with V10 ≤ 50% and 29.2% in patients with V10 > 50%, respectively. Considering that, we want to point out V10 of the lungs for patients with neoadjuvant treatment. While mean V10 was 52.6% when 4 cm longitudinal margins were used, mean V10 was 48.4% and 42.7% for longitudinal margins of 3 cm and 2 cm. This is another hint that risk of radiation pneumonitis might be significantly decreased by using smaller longitudinal margins.

Considering SCC patients one has to keep in mind that these patients often receive concomitant chemotherapy with paclitaxel. This is important because chemotherapy with taxanes is a known risk factor for pneumonitis [39] and the time period between radiation and beginning of taxan therapy was associated with the risk of pneumonitis in patients with metastatic or recurrenct EC after radiation therapy [40]. Because concomitant chemotherapy is not considered by the NTCP-model, the calculated risk of pneumonitis is probably underestimated.

It is well known that modern radiation techniques like VMAT can improve dose conformity as well as homogeneity and also can decrease the OAR doses compared to only 3D conformal radiotherapy [41–45]. However, in this study all treatment plans were calculated for VMAT, which might also explain the low OAR doses and the resulting low risk of side effects.

Nevertheless, our data demonstrate that all relevant dose parameters for the lungs could significantly be reduced when comparing a longitudinal margin of 3 cm to a longitudinal margin of 4 cm. In addition there was a strong trend towards a significant reduction of the risk to develop a radiation pneumonitis (*p* = 0.059).

When talking about calculating the risk of side effects by using NTCP models it is important to notice, that especially for patients undergoing neoadjuvant chemoradiation and subsequent surgery the risk of side effects not only depends on radiation dose or chemotherapy. Instead, nCRT is known to increase the risk of postoperative pulmonary complications. In a study by Wang et al. [46] V5 was an independent predictor of postoperative pulmonary complications. In our analysis, the mean V5 was reduced by 5% when longitudinal margins were reduced to 3 cm. Therefore, NTCP models alone are probably insufficient to calculate the risk of side effect. However, smaller longitudinal margins might also lead to a reduced incidence of postoperative pulmonary complications.

Regarding the heart, previous studies identified Dmean, V30 and Dmedian as relevant parameters to predict the risk of pericardial effusion after RT [47–49]. Of these, at least Dmean was significantly reduced by shorter longitudinal margins. As for the lungs, dose parameters as well as the resulting risk for pericardial effusion were very low compared to the literature. We think that this is the reason why no difference in the risk of pericardial effusion was seen, even when reducing the longitudinal margin to 2 cm.

Another aspect is that smaller CTVs might give us the opportunity to increase tumor control by using higher radiation doses without increasing toxicity. However, when thinking about dose escalation we have to keep in mind that the CROSS trial [10] revealed excellent results for nCRT with a relatively small radiation dose while the best dose concept for dCRT is still a matter of debate.

## Conclusion

Reduction of longitudinal margins leads to significant lower OAR doses, especially for the lungs. However, absolute differences are small and clinical relevance is difficult to assess. Nevertheless, shorter longitudinal margins seem to be feasible for SCC and prospective trials involving modern diagnostic imaging should evaluate clinical outcome.

## Abbreviations

AAA: Anisotropic analytical algorithm
CRT: Chemoradiation
CTV: Clinical target volume
dCRT: Definite chemoradiation
Dmax: Maximum dose
Dmean: Mean dose
Dmedian: Median dose
Dmin: Minimum dose
DVH: Dose-volume histogram
EC: Esophageal cancer
ENI: Elective nodal irradiation
GTV: Gross target volume
IQR: Interquartile range
ITV: Internal target volume
nCRT: Neoadjuvant chemoradiation
NTCP: Normal tissue complication probability
OAR: Organs at risk
PET: Positron emission tomography
PTV: Planning target volume
RT: Radiotherapy
SCC: Squamous cell carcinoma of the esophagus
VMAT: Volumetric-modulated arc therapy
Vx: Volume (of the whole organ) receiving more than x Gray
